# Aetiologies of Central Nervous System Infection in Viet Nam: A Prospective Provincial Hospital-Based Descriptive Surveillance Study

**DOI:** 10.1371/journal.pone.0037825

**Published:** 2012-05-25

**Authors:** Nghia Ho Dang Trung, Tu Le Thi Phuong, Marcel Wolbers, Hoang Nguyen Van Minh, Vinh Nguyen Thanh, Minh Pham Van, Nga Tran Vu Thieu, Tan Le Van, Diep To Song, Phuong Le Thi, Thao Nguyen Thi Phuong, Cong Bui Van, Vu Tang, Tuan Hoang Ngoc Anh, Dong Nguyen, Tien Phan Trung, Lien Nguyen Thi Nam, Hao Tran Kiem, Tam Nguyen Thi Thanh, James Campbell, Maxine Caws, Jeremy Day, Menno D. de Jong, Chau Nguyen Van Vinh, H. Rogier Van Doorn, Hien Tran Tinh, Jeremy Farrar, Constance Schultsz, the VIZIONS CNS Infection Network

**Affiliations:** 1 Pham Ngoc Thach University of Medicine, Ho Chi Minh City, Viet Nam; 2 Centre for Tropical Medicine, Oxford University Clinical Research Unit, Ho Chi Minh City, Viet Nam; 3 Hospital for Tropical Diseases, Ho Chi Minh City, Viet Nam; 4 Dong Thap Provincial Hospital, Dong Thap Province, Viet Nam; 5 An Giang Provincial Hospital, An Giang Province, Viet Nam; 6 Kien Giang Provincial Hospital, Kien Giang Province, Viet Nam; 7 Soc Trang Provincial Hospital, Soc Trang Province, Viet Nam; 8 DakLak Provincial Hospital, DakLak Province, Viet Nam; 9 Khanh Hoa Provincial Hospital, Khanh Hoa Province, Viet Nam; 10 Hue Central Hospital, Thua Thien – Hue Province, Viet Nam; 11 Can Tho Central Hospital, Can Tho City, Viet Nam; 12 Centre for Tropical Medicine, Oxford University, Oxford, United Kingdom; 13 Department of Global Health, Academic Medical Center, University of Amsterdam, Amsterdam, The Netherlands; 14 Department of Medical Microbiology, Academic Medical Center, University of Amsterdam, Amsterdam, The Netherlands; Centers for Disease Control and Prevention, United States of America

## Abstract

**Background:**

Infectious diseases of the central nervous system (CNS) remain common and life-threatening, especially in developing countries. Knowledge of the aetiological agents responsible for these infections is essential to guide empiric therapy and develop a rational public health policy. To date most data has come from patients admitted to tertiary referral hospitals in Asia and there is limited aetiological data at the provincial hospital level where most patients are seen.

**Methods:**

We conducted a prospective Provincial Hospital-based descriptive surveillance study in adults and children at thirteen hospitals in central and southern Viet Nam between August 2007– April 2010. The pathogens of CNS infection were confirmed in CSF and blood samples by using classical microbiology, molecular diagnostics and serology.

**Results:**

We recruited 1241 patients with clinically suspected infection of the CNS. An aetiological agent was identified in 640/1241 (52%) of the patients. The most common pathogens were *Streptococcus suis* serotype 2 in patients older than 14 years of age (147/617, 24%) and Japanese encephalitis virus in patients less than 14 years old (142/624, 23%). *Mycobacterium tuberculosis* was confirmed in 34/617 (6%) adult patients and 11/624 (2%) paediatric patients. The acute case fatality rate (CFR) during hospital admission was 73/617 (12%) in adults and to 42/624 (7%) in children.

**Conclusions:**

Zoonotic bacterial and viral pathogens are the most common causes of CNS infection in adults and children in Viet Nam.

## Introduction

Despite advances in antibiotic treatment and resuscitation, infectious diseases of the central nervous system (CNS) remain life-threatening, especially in developing countries. According to the World Health Organization (WHO), there were approximately 700,000 episodes of meningitis in 2004 and 70% of these patients lived in Africa and South-East Asia [1]. Every year, Japanese encephalitis virus, an important cause of encephalitis in Asia, causes 50,000 encephalitis cases and 15,000 deaths and leaves many survivors with severe neurological and psychiatric sequelae, whilst the case fatality rate in treated patients with tuberculous meningitis (TBM) is 65% in HIV-infected patients and 25% in HIV uninfected patients in Viet Nam [2], [3]. To reduce the morbidity and mortality of CNS infections, studies are needed to establish the aetiology in order to design rational policies for prevention and empiric and specific treatment. The cause of CNS infection may vary over time, by geographic region, with age, co-morbidities, vaccination programs and the routes by which the pathogens are acquired [4]–[6]. However, the epidemiological data related to the aetiology of CNS infections in Viet Nam are limited and come from tertiary referral hospitals in major cities. The identification of the causes of CNS infections in Viet Nam is difficult for many reasons, including limited bacteriological culture facilities, lack of PCR and viral culture and the widespread use of antibiotics prior to presentation to any hospital. Hence, we conducted a prospective provincial hospital-based surveillance study to identify the causes of CNS infections in central and southern Viet Nam.

## Materials and Methods

### Ethical Approval

This study was approved by the Scientific and Ethics Committee of each study site (provincial hospitals), the Hospital for Tropical Diseases and the University of Oxford Tropical Research Ethics Committee (OXTREC 01–08). Informed consent was obtained or proxy consent from a relative was obtained for a patient with GCS score <15 or a patient under 15 year-old on admission. Patients were excluded if they did not provide written informed consent.

### Study Design and Setting

**Figure 1:**
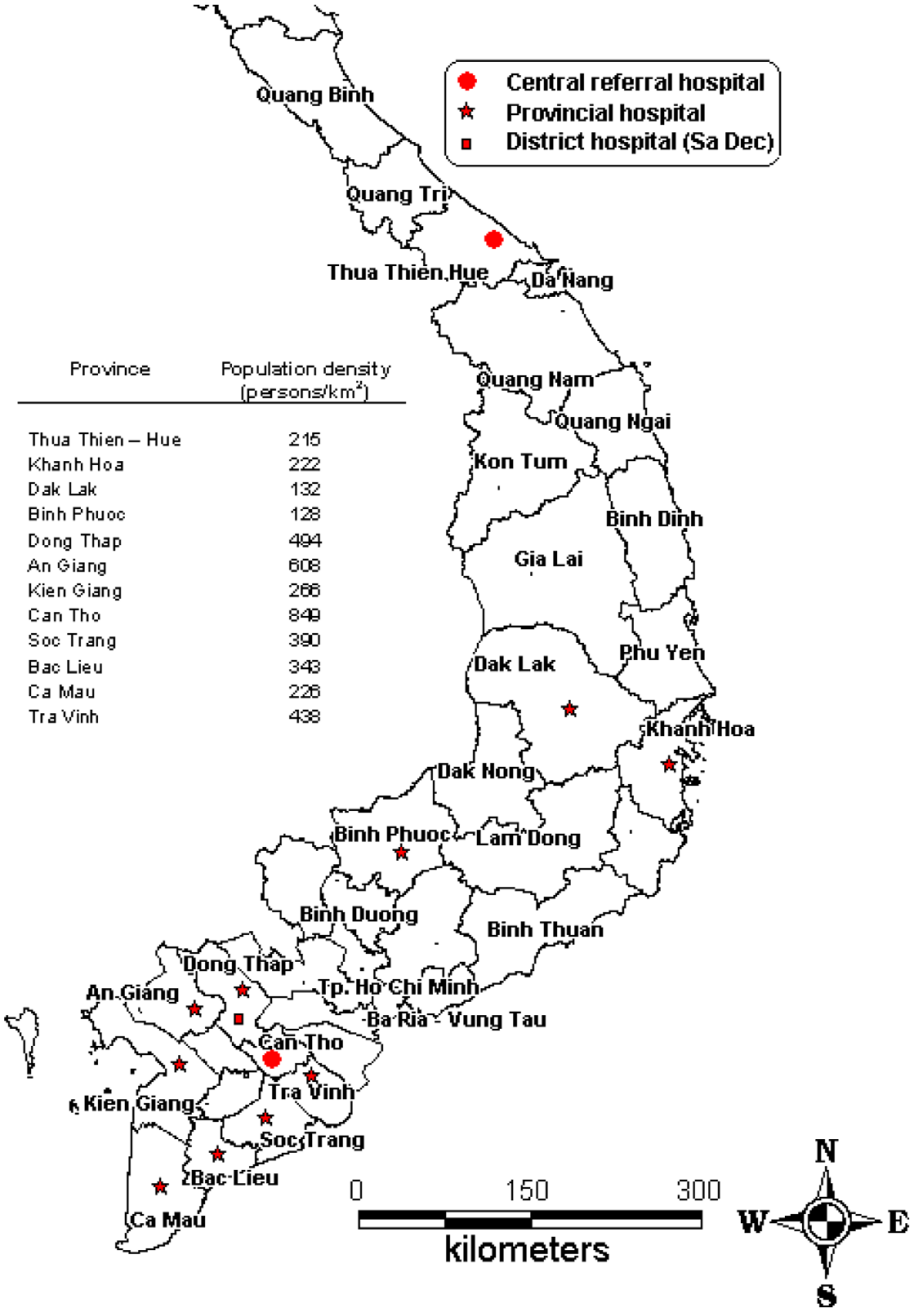
Locations of study sites. Map of southern Vietnam indicating participating hospitals and population density in the catchment area of each site**.**

A prospective hospital-based descriptive surveillance study was conducted at thirteen hospitals in central and southern Viet Nam, including one district hospital (Sa Dec, Dong Thap), seven provincial hospitals (Dong Thap, An Giang, Kien Giang, Ca Mau, Bac Lieu, Soc Trang, Tra Vinh) and one central referral hospital (Can Tho) in the Mekong River Delta; one provincial hospital (Binh Phuoc) in the South East; one provincial hospital (Dak Lak) in the Central Highlands; and one provincial hospital (Khanh Hoa) and one central referral hospital (Thua Thien-Hue) in central Viet Nam (Figure 1). The study was conducted between August 2007 and April 2010.

### Participants and Recruitment

Patients who presented at the infectious diseases ward, intensive care unit or paediatric ward of participating hospitals, were eligible if they met all of the following inclusion criteria; at least one month of age; fever ≥ 38°C (axillary); at least one of the following symptoms or signs: headache, neck stiffness, altered consciousness and focal neurological signs; and a cerebrospinal fluid (CSF) sample taken. Patients were excluded if they or their relatives did not provide written informed consent. The attending physician examined the patients and completed a standardized clinical data form. Data collected included demographics, history and examination findings, past medical history, laboratory findings, treatment and outcome. Four milliliters of CSF were taken from each adult patient and 2 mls from children 5 years of age or older and 1 ml from children under five. Cerebrospinal fluid was sent to the hospital laboratory for cell count, biochemistry measurements and microbiology investigations according to the local standard of care. A half to one milliliter was stored at –20°C and later transferred to the Hospital for Tropical Diseases in Ho Chi Minh City (HTD) on dry ice for further investigations, including 200 µl for diagnostic PCR (bacteria and virus) and 100 µl for JE and Dengue serology. All bacterial isolates from CSF and blood cultures had identification confirmed at HTD and antimicrobial susceptibility assessment.

### Definitions

#### Adults and children

Patients were assigned as adults if they were older than or equal to 15 years of age on the day of admission according to Vietnamese law. If they were less than 15 years of age, they were assigned as children.

#### Case definitions of clinical syndromes

Clinical syndromes were defined in accordance with WHO case definitions of bacterial meningitis (BM) and viral encephalitis (VE), and using a consensus case definition of TBM [7], [8]. If CSF lactate was available, it was included in the case definition of BM and viral encephalitis/meningitis (VE/M) (see Table 1). A patient was considered to not have a CNS infection if they had normal CSF parameters with no pathogen identified, and if the clinical diagnosis at discharge was non-infectious such as stroke, epilepsy, psychiatric disorder, drug or alcohol toxicity, hepatic encephalopathy, sepsis, or benign febrile convulsion.

**Table 1:** Case definitions of clinical syndromes.

Bacterial meningitis (modified from case definition of BM of WHO [7])	
***Laboratory-confirmed*** (at least one of the following criteria)	***Probable***
-Positive culture, Gram stain or real-time PCR of CSF sample	-Sudden onset of fever (>38°C) (less than 7 days)
-Positive bacterial blood culture and clinical syndrome consistent with BM (see probable criteria)	-AND at least one of the following signs:
	oMeningeal signs (neck stiffness, Kernig sign and Brudzinski sign)
	OAltered consciousness
	-AND CSF examination showing at least one of the following:
	oLeukocytosis (≥10 cells/µl) AND at least 2 of the following criteria^1^:
	•an elevated protein (>1 g/l)
	•decreased glucose (<2.2 mmol/l or less than 50% of blood glucose)
	•lactate ≥4 mmol/l
	OTurbid appearance (when WC is missing or WC <10/µl)
	-AND no aetiologic agent was identified
**Viral encephalitis/meningitis (modified from case definition of acute encephalitis syndrome of WHO ** [**7**] **)**	
*Laboratory-confirmed*	***Probable***
-Laboratory confirmation by real-time PCR (enterovirus and *Herpes simplex*) or MAC ELISA (Japanese Encephalitis virus) on CSF	-Acute onset of fever (less than 7 days)
-A case was classified as probable Japanese encephalitis (JE) if a patient had the following criteria:	-AND at least one of the following:
oFulfilled the case definition of probable VE/M	OMeningeal signs (neck stiffness, Kernig sign, and Brudzinski sign)
oJEV specific IgM titre in CSF was in the range from 8–12 U	oChange in mental status (confusion, disorientation, coma or inability to talk)
oNo other pathogen detected.	oNew onset of seizures (excluding simple febrile seizures)
-A case was classified as Dengue encephalitis/meningitis, if Dengue virus specific IgM titre in CSF was higher than 12 U	-AND CSF examination showing at least one of the following:
-A case was classified as possible Dengue encephalitis/meningitis if patient had the following criteria:	OLeukocytosis (≥10 cells/µl) AND at least 2 of these criteria^2^:
oFulfilled case definition of probable VE/M	•protein ≤1 g/l
oDengue virus specific IgM titre in CSF was in the range from 8–12 U	•normal glucose (≥2.2 mmol/l or ≥50% of blood glucose)
oNo other pathogen detected.	•lactate <4 mmol/l
	OClear appearance (when WC is missing or WC <10/µl)
	-AND no aetiologic agent was identified
**Tuberculous meningitis ** [**8**]	
***Laboratory-confirmed***	***Probable***
Positive smear (acid-fast bacilli, AFB) or real-time PCR of CSF	-Total diagnostic score ≥10 (when cerebral imaging is not available) or ≥12 (when cerebral imaging is available)
	-At least 2 points should either come from CSF or cerebral imaging criteria.
	***Possible***
	-Total diagnostic score of 6–9 points (when cerebral imaging is not available) or 6–11 points (when cerebral imaging is available)
	-Possible tuberculosis cannot be diagnosed or excluded without doing a lumbar puncture or cerebral imaging.
**Cryptococcal meningitis**	
An India ink stain of CSF positive showing encapsulated yeasts, or*Cryptococcus neoformans* cultured from CSF.	
**Eosinophilic meningitis**	
***Confirmed***	***Probable***
Meningitis and a percentage of eosinophils in CSF greater than 10% [63]	-Percentage of eosinophilic cells in blood >10%
	-AND meningitis manifestations
	-AND no pathogen confirmed in CSF by culture, PCR or ELISA method

1 If lactate concentration was not available, a patient was diagnosed as BM when having at least one of the remaining criteria.

2 If lactate concentration was not available, a patient was diagnosed as VE/M when having the two remaining criteria.

### Microbiological Investigations

#### Blood and CSF culture

Blood and CSF cultures were performed in the microbiology laboratory of the hospitals using standard culture methods. Blood culture was performed using a BD BACTEC® 9050 blood culture system (BD Microbiology, USA) in Dong Thap and Kien Giang hospitals, and by the BacT/Alert blood culture system (bioMérieux, France) in Hue Central hospital. Manual blood culture using commercially available blood culture media purchased in Vietnam (Nam Khoa Biotek, Ho Chi Minh city, Viet Nam) were used in the remaining hospitals. CSF cultures were incubated overnight in candle jars, except in Hue Central hospital where a CO_2_ incubator was available. Laboratories used the locally available tools for identification of isolates from positive cultures. In addition, all bacterial isolates were sent to HTD for bacterial identification and antimicrobial susceptibility testing.

#### Molecular methods

Internally controlled real-time PCR was used for the detection of *Streptococcus pneumoniae*, *Haemophilus influenzae* type b, *Neisseria meningitidis*, *Streptococcus suis* serotype 2, *Herpes simplex virus* 1 and 2, enteroviruses (generic) and *Mycobacterium tuberculosis* in CSF samples using methods published previously [9]–[12]. The RealAccurate™ *Mycobacterium tuberculosis* PCR kit (Patho Finder, Maastricht, The Netherlands), targeting the IS6110 sequence, was used to confirm the clinical diagnosis of TBM. This PCR kit is validated for use on respiratory samples. Its sensitivity, specificity, positive predictive value and negative predictive value were evaluated at the HTD laboratory using an appropriate set of CSF samples, including 24 TBM CSF samples confirmed by CSF smear (AFB) or mycobacterial growth indicator tube (MGIT) culture method, 16 BM CSF samples confirmed by culture and 2 *Herpes simplex* encephalitis CSF samples confirmed by PCR and were 67%, 94%, 94% and 68%, respectively (unpublished data).

**Figure 2:**
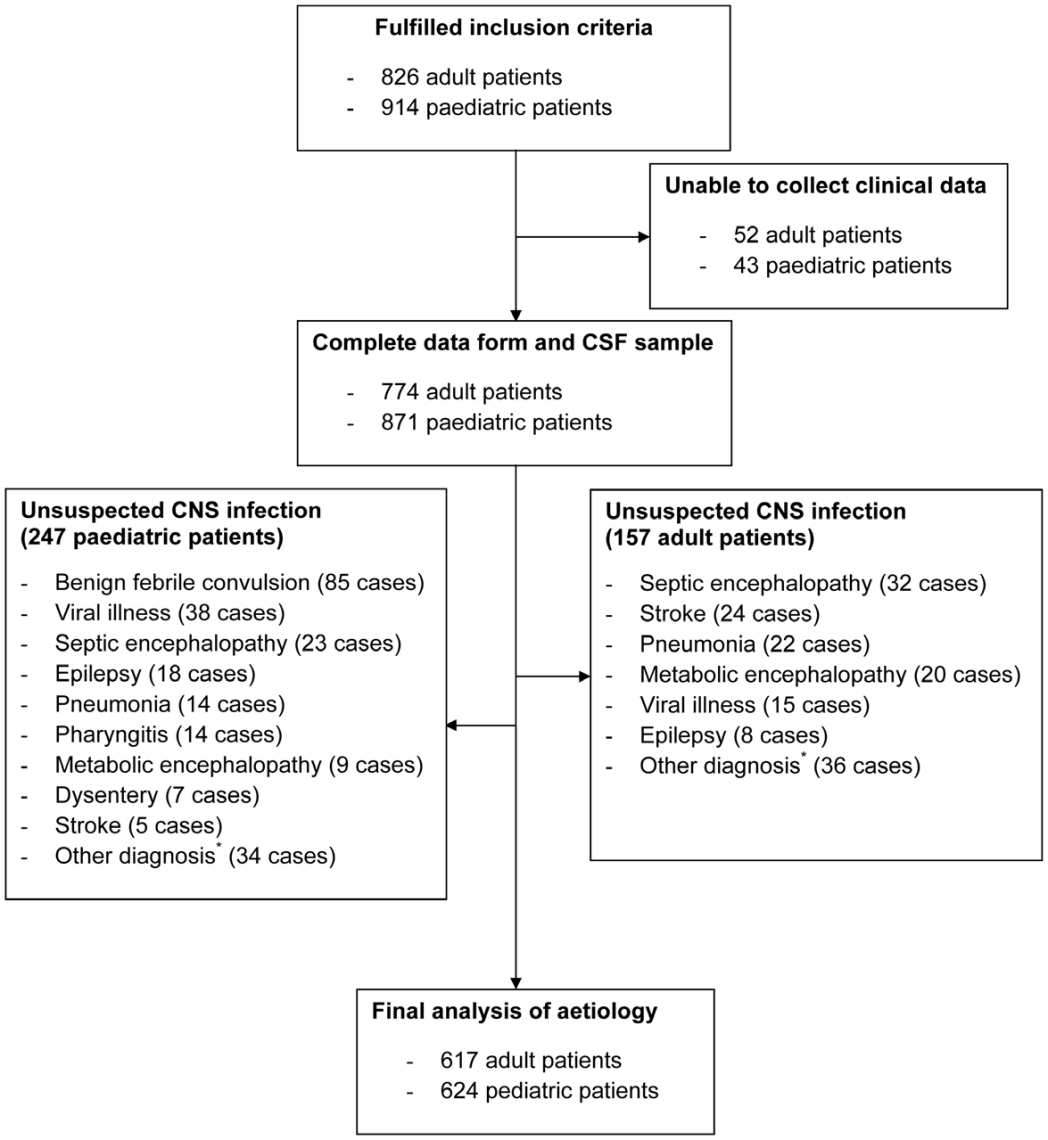
Study profile. (*) Other diagnosis included sinusitis (5 cases), brain tumours (9 cases), cerebral malaria (8 cases), mental disorders (7 cases), headache (7 cases), typhoid fever (5 cases), fever unknown origin (3 cases), autoimmune diseases (12 cases), diarrhoea (6 cases), lumbar disc herniation (2 cases), congenital heart diseases (2 cases), hydrocephalus (1 case), tetanus (1 case), severe anaemia (1 case) and chronic colitis (1 case).

#### Serological methods (CSF samples)

To confirm infection with JE virus or Dengue virus, a JEV/DENV IgM ELISA assay (Venture Technologies Sdn Bhd Malaysia), was used. This assay uses antigens from JEV and DENV1-DENV4 to demonstrate and distinguish recent infection by these two flaviviruses [13].

**Table 2:** Demographic data, clinical diagnosis and outcome of patients with CNS infection enrolled in the study.

Characteristics	Adults (n = 617)	Children (n = 624)	p value^3^
**Median age, years (IQR)**	38 (24; 52)	4 (1.29; 9)	_
**Male, n (%)**	431 (70)	385 (62)	0.002
**Rural, n (%)**	502 (81)	527 (85)	0.1
**Regions, n (%)**			<0.001
-Mekong river delta	353 (57)	309 (49)	
-Central Viet Nam	211 (34)	204 (33)	
-Central highlands	38 (6)	104 (17)	
-South East	10 (2)	6 (1)	
-Others^1^	5 (1)	1 (0)	
**Ethnicity, n (%)**			<0.001
-Kinh	528 (86)	478 (77)	
-Khmer	64 (10)	68 (11)	
-E de	10 (1.5)	27 (4)	
-Raglai	3 (0.5)	14 (2)	
-Others^2^	12 (2)	37 (6)	
**HIV status, n (%)**			
-Positive	10 (2)	0 (0)	-
-Negative	488 (79)	50 (8)	-
-Unknown	119 (19)	574 (92)	-
**Diagnosis, n (%)**			<0.001
-Bacterial meningitis	302 (49)	150 (24)	
oConfirmed cases	198 (32)	91 (15)	
oProbable cases	104 (17)	59 (9)	
-Viral encephalitis/meningitis	209 (34)	432 (69)	
oConfirmed cases	76 (12)	206 (33)	
oProbable cases	133 (22)	226 (36)	
-Tuberculous meningitis	87 (14)	31 (5)	
oConfirmed cases	34 (5)	11 (1.7)	
oProbable cases	10 (2)	2 (0.3)	
oPossible cases	43 (7)	18 (3)	
-Eosinophilic meningitis	4 (0.6)	1 (0.1)	
-Cryptococcal meningitis	2 (0.3)	0 (0)	
-Cerebral toxoplasmosis	1 (0.1)	0 (0)	
-Dual infection	12 (2)	10 (2)	
**Outcomes, n (%)**			<0.001
-Alive	429 (70)	493 (79)	
-Died	73 (12)	42 (7)	
-Transferred to other hospitals	88 (14)	71 (11)	
-Unknown outcome	27 (4)	18 (3)	

1 Cambodia, Laos.

2 Cham, Co Tu, Dao, H’Mong, Jarai, M’Nong, Mong, Nung, Pa Ko, STieng, Ta Oi, Tay, Thai, Xo Dang, Van Kieu, Chinese and Laotian.

3 Chi-squared test or Fisher’s exact test (when one or more of the expected count is less than 5).

### Analysis

All variables were summarized by group (adult or children). Patients were assigned to confirmed or probable BM, VE/M or TBM according to the pre-specified case definitions of clinical syndromes (Table 1). Epidemiological characteristics of patients were analyzed for all patients with confirmed or probable CNS infections. Categorical variables were summarized as numbers and percentages (%). Continuous variables were summarized as median and interquartile range (IQR). Categorical baseline variables were compared between adults and children using chi-squared test or Fisher’s exact test (when one or more of the expected cell counts was less than 5). Time trends and seasonality of overall admission numbers of children and adults with CNS infections as well as seasonality of the number of *S. suis* meningitis cases was assessed using Poisson regression models, which modelled the monthly admission counts depending on a hospital effect, a global linear time trend and a sinusoidal shape for the seasonality effect (modelled with a sine- and a cosine-term, i.e. a cosinor model) [14]. The log-number of days per month was used as an offset in the model to correct for unequal lengths of the month. For testing seasonality we tested whether the sine- and the cosine-term could jointly be omitted from the model using a likelihood ratio test. All analyses were performed with Stata version 10.1 (StataCorp, College Station, Texas, USA) and R version 2.11.1 (R Foundation for Statistical Computing, Vienna, Austria) software.

**Figure 3:**
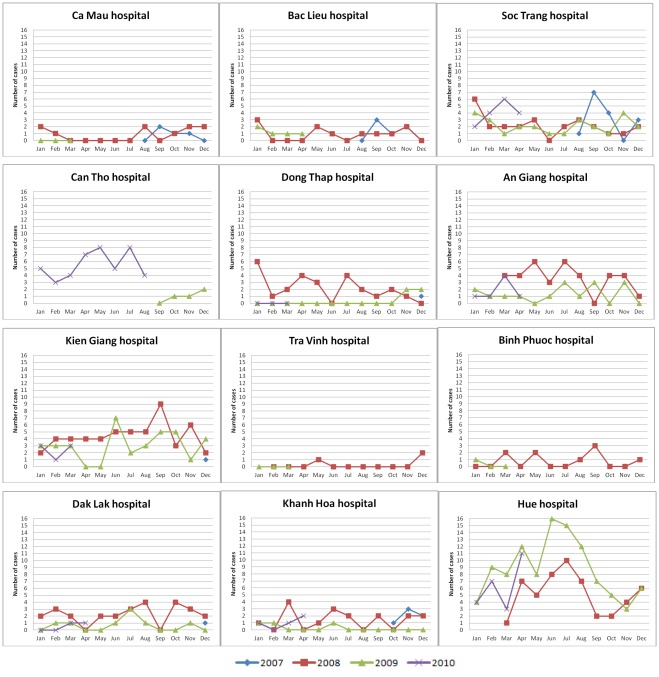
Time distribution of adult CNS infection admissions per study site. Number of cases: absolute number of adult patients with CNS infection enrolled in the study per month.

**Figure 4:**
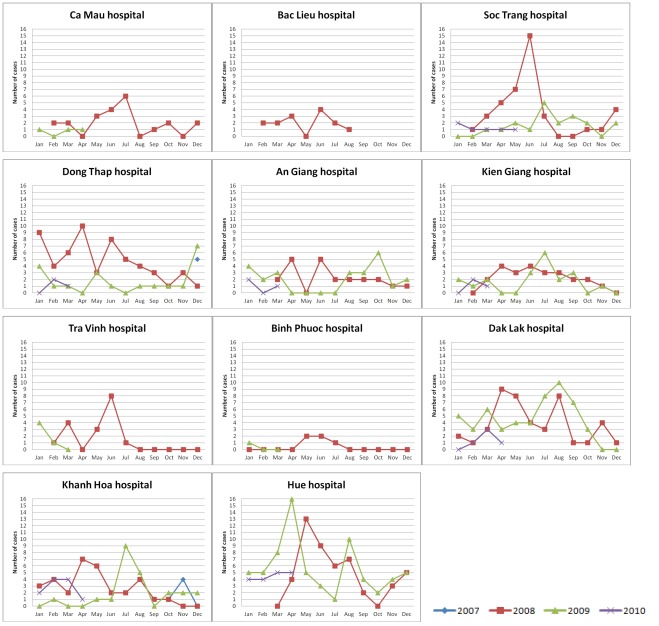
Time distribution of paediatric CNS infection admission per study site. Number of cases: absolute number of children with CNS infection enrolled in the study per month.

**Figure 5:**
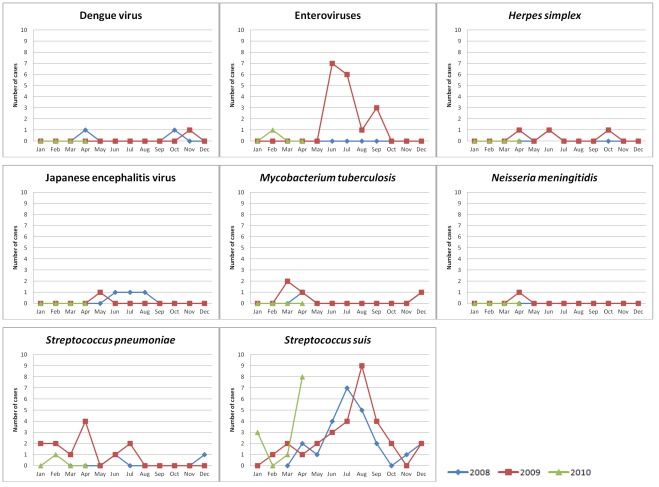
Time distribution of adult CNS infection admissions by all major pathogens at Hue Central hospital. Number of cases: number of adult patients with CNS infection enrolled in the study at Hue Central hospital per month for 8 pathogens (results of PCR, serology and bacterial culture combined).

## Results

Between August 2007 and April 2010, 1740 patients fulfilling the inclusion criteria were admitted to 2 central, 10 provincial and 1 district hospitals. None refused to provide consent. Clinical data were not available for 95 patients, including 52 adults and 43 children. In these 95 patients, pathogens were confirmed in 12/52 adults (eight *S. suis* serotype 2, one *S. pneumoniae*, one JE virus, one enterovirus and one co-infection with *S. suis* and enterovirus) and in 13/43 children (four enteroviruses, two JE viruses, two dengue viruses, two *H. influenzae*, one *N. meningitidis* and two co-infections with *S. pneumoniae* and JE virus). After reviewing the clinical presentation and hospital tests of 1645 patients that had complete clinical data and a CSF sample taken, we excluded 247 paediatric and 157 adult patients in the final analysis of the causative aetiology (Figure 2). These patients all had normal CSF data and a clinical history consistent with a non-infectious cause. Thus 1241 patients (617 adults, 624 children) were included in the final analysis.

**Table 3:** Laboratory confirmed aetiology for adults with CNS infection enrolled in the study.

Pathogen, n (%)	Adults (n = 617)		
	PCR or serology results	Bacterial culture results	Combined results of PCR, serology and culture
**Bacteria**			
*Streptococcus suis* serotype 2	147 (24)	99 (16)	147 (24)
*Streptococcus pneumoniae*	35 (6)	15 (2)	35 (6)
*Haemophilus influenzae* type b	0 (0)	0 (0)	0 (0)
*Neisseria meningitidis*	3 (0.5)	1 (0.1)	4 (0.6)
*Streptococcus spp*	–	2 (0.3)	2 (0.3)
*Staphylococcus spp*	–	1 (0.1)	1 (0.1)
*Escherichia coli*	–	2 (0.3)	2 (0.3)
*Acinetobacter spp*	–	1 (0.1)	1 (0.1)
*Klebsiella pneumoniae*	–	5 (0.8)	5 (0.8)
*Enterococcus spp*	–	1 (0.1)	1 (0.1)
**Virus**			
Japanese Encephalitis (JE) virus	11 (2)	–	11 (2)
Dengue virus	23 (4)	–	23 (4)
Enteroviruses	20 (3)	–	20 (3)
*Herpes simplex*	22 (4)	–	22 (4)
**Tuberculosis**			
*Mycobacterium tuberculosis*	34 (5)	–	34 (5)
**Fungi**			
*Cryptococcus neoformans*	2 (0.3)	–	2 (0.3)
**Dual infection**			
Dengue virus + *S. suis* serotype 2	2 (0.3)	1 (0.1)	2 (0.3)
Dengue virus + *S. pneumoniae*	1 (0.1)	0 (0)	1 (0.1)
Dengue virus + *N. meningitidis*	1 (0.1)	0 (0)	1 (0.1)
Dengue virus + *M. tuberculosis*	2 (0.3)	–	2 (0.3)
Dengue virus + Eosinophilic meningitis^1^	2 (0.3)	–	2 (0.3)
JE virus + *S. pneumoniae*	1 (0.1)	0 (0)	1 (0.1)
Enteroviruses + *M. tuberculosis*	1 (0.1)	–	1 (0.1)
*K. pneumoniae* + *M. tuberculosis*	–	1 (0.1)	1 (0.1)
*K. pneumoniae* + *Herpes simplex*	–	1 (0.1)	1 (0.1)
Unknown aetiology^2^	310 (50)	487 (79)	295 (48)

1 Number of eosinophils in CSF sample was 352/880 (40%) in one case and 330/1320 (25%) in another case.

2 “Unknown aetiology” corresponds to the probable cases.

**Table 4:** Laboratory confirmed aetiology for children with CNS infection enrolled in the study.

Pathogen, n (%)	Children (n = 624)		
	PCR or serology results	Bacterial culture results	Combined results of PCR, serology and culture
**Bacteria**			
*Streptococcus suis* serotype 2	0 (0)	0 (0)	0 (0)
*Streptococcus pneumoniae*	36 (6)	16 (3)	37 (6)
*Haemophilus influenzae* type b	39 (6)	9 (1)	39 (6)
*Neisseria meningitidis*	6 (1)	2 (0.3)	6 (1)
*Staphylococcus spp*	–	3 (0.5)	3 (0.5)
*Escherichia coli*	–	2 (0.3)	2 (0.3)
*Acinetobacter spp*	–	1 (0.1)	1 (0.1)
*Klebsiella pneumoniae*	–	1 (0.1)	1 (0.1)
*Salmonella spp*	–	2 (0.3)	2 (0.3)
**Virus**			
Japanese Encephalitis (JE) virus	142 (23)	–	142 (23)
Dengue virus	14 (2)	–	14 (2)
Enteroviruses	36 (6)	–	36 (6)
*Herpes simplex*	14 (2)	–	14 (2)
**Tuberculosis**			
*Mycobacterium tuberculosis*	11 (2)	–	11 (2)
**Dual infection**			
Dengue virus + *H. influenzae* type b	3 (0.5)	1 (0.1)	3 (0.5)
JE virus + *N.meningitidis*	1 (0.1)	0 (0)	1 (0.1)
JE virus + *H. influenzae* type b	3 (0.5)	0 (0)	3 (0.5)
JE virus + *Salmonella spp*	–	1 (0.1)	1 (0.1)
JE virus + *Staphylococcus spp*	–	1 (0.1)	1 (0.1)
Enteroviruses + *H. influenzae* type b	1 (0.1)	0 (0)	1 (0.1)
Unknown aetiology^1^	318 (51)	585 (94)	306 (49)

1 “Unknown aetiology” corresponds to the probable cases.

### Epidemiological Characteristics of CNS Infection

Over 80% of CNS infection patients lived in rural areas and belonged to the Kinh ethnic group. The proportion of ethnic minorities amongst children was higher than that in adults (23% versus 14%, p<0.001) (Table 2). The ratio of males to females was 1.92/1.00. The aetiology of CNS infection in children was different to that in adults. VE/M were responsible for 432/624 (70%) of paediatric CNS infections, compared to 209/617 (34%) of adult infections. Acute BM and TBM were more common in the adults (Table 2). The overall case fatality rate (CFR) at provincial hospitals was 73/617 (12%) in adults compared to 42/624 (7%) in children (Table 2). Of patients with TBM, 24/87 (28%) adult patients and 12/31 (39%) paediatric patients died before receiving anti-tuberculosis drugs.

The number of patients admitted to each of the participating hospitals and their distribution in time is depicted in Figure 3 and 4. Analysis of admission patterns of adult patients with CNS infections by Poisson regression revealed on overall linear decline in the incidence by −13% per year (95%CI [−23%; −1%]) and a significant seasonality effect (p<0.01) indicating peak incidence in June and a difference between the peak vs. the average incidence of +21% (95%CI [+8%; 36%]). However, the linear trend failed to reach conventional significance after adjustment for overdispersion (p = 0.06). Moreover, the seasonal pattern was driven by Hue Central hospital alone - the test for seasonality was significant there (p<0.001) but did not reach significance in all other hospitals combined (p = 0.36) (Figure 3). The seasonality at Hue Central hospital was driven by *S. suis* infection (Figure 5).

**Table 5:** Laboratory confirmed aetiology for adult patients meeting the case definition of bacterial meningitis enrolled in the study (excluding dual infections).

Pathogen, n (%)	Adults (n = 302)		
	PCR results	Culture results	Combined results of PCR and culture
*Streptococcus suis* serotype 2	147 (49)	99 (33)	147 (49)
*Streptococcus pneumoniae*	35 (12)	15 (5)	35 (12)
*Haemophilus influenzae* type b	0 (0)	0 (0)	0 (0)
*Neisseria meningitidis*	3 (1)	1 (0.1)	4 (1)
*Streptococcus spp*	–	2 (0.6)	2 (0.6)
*Staphylococcus spp*	–	1 (0.3)	1 (0.3)
*Escherichia coli*	–	2 (0.6)	2 (0.6)
*Acinetobacter spp*	–	1 (0.3)	1 (0.3)
*Klebsiella pneumoniae*	–	5 (2)	5 (2)
*Enterococcus spp*	–	1 (0.3)	1 (0.3)
Unknown aetiology^1^	117 (38)	175 (58)	104 (34)

1 “Unknown aetiology” corresponds to the probable cases.

**Table 6:** Laboratory confirmed aetiology for paediatric patients meeting the case definition of bacterial meningitis enrolled in the study (excluding dual infections).

Pathogen, n (%)	Children (n = 150)		
	PCR results	Culture results	Combined results of PCR and culture
*Streptococcus suis* serotype 2	0 (0)	0 (0)	0 (0)
*Streptococcus pneumoniae*	36 (24)	16 (11)	37 (25)
*Haemophilus influenzae* type b	39 (26)	9 (6)	39 (26)
*Neisseria meningitidis*	6 (4)	2 (1)	6 (4)
*Staphylococcus spp*	–	3 (2)	3 (2)
*Escherichia coli*	–	2 (1)	2 (1)
*Acinetobacter spp*	–	1 (1)	1 (1)
*Klebsiella pneumoniae*	–	1 (1)	1 (1)
*Salmonella spp*	–	2 (1)	2 (1)
Unknown aetiology^1^	69 (46)	114 (76)	59 (39)

1 “Unknown aetiology” corresponds to the probable cases.

**Figure 6:**
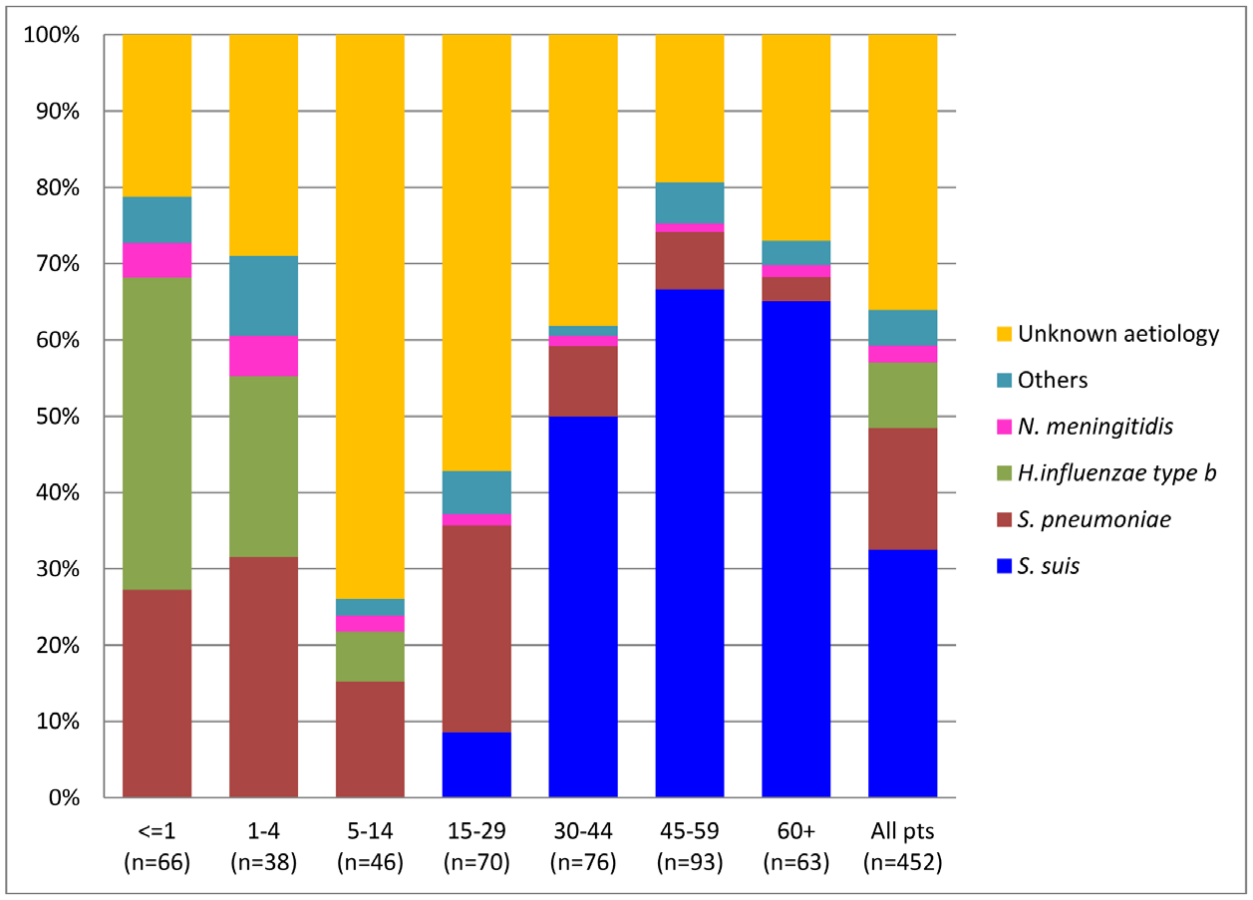
Pathogens of bacterial meningitis by age group (excluding dual infection cases). Laboratory confirmed aetiology per agegroup (bars) for patients meeting the case definition of bacterial meningitis. “Unknown aetiology” corresponds to patients with a diagnosis of probable bacterial meningitis.

A seasonal pattern of paediatric CNS infection admissions was also noted. Analysis of admission patterns by Poisson regression showed a significant seasonality effect (p<0.001). The admissions to hospitals peaked in June with an amplitude of difference (peak month vs. average) of +51% (95%CI [+34%; +69%]) and a linear time trend of −24% per year (95% CI [−33%; −14%], p<0.001) (Figure 4).

### Aetiology of CNS Infection

An aetiological agent was identified in 640/1241 (52%) of patients (Table 3 and 4). The most common pathogens were *Streptococcus suis* serotype 2 in adults (147/617, 24%) and JE virus in children (142/624, 23%). *Mycobacterium tuberculosis* was confirmed by PCR in 34/617 (6%) of adults and 11/624 (2%) of children. Dual infection was demonstrated in 22 cases (12 adults, 10 children). Dual infection was most commonly due to dengue virus accompanying a bacterial pathogen (9/22 cases) or JE virus and a bacterial pathogen (7/22 cases).

**Table 7:** Laboratory confirmed aetiology for patients meeting the case definition of viral encephalitis/meningitis enrolled in the study (excluding dual infections).

Pathogen, n (%)	Adults (n = 209)	Children (n = 432)	p value^1^
JE virus	11 (5)	142 (33)	<0.001
Dengue virus	23 (11)	14 (3)	<0.001
Enteroviruses	20 (10)	36 (8)	0.603
*Herpes simplex*	22 (11)	14 (3)	<0.001
Unknown aetiology^2^	133 (64)	226 (52)	0.007

1 Chi-squared test or Fisher’s exact test (when one or more of the expected count is less than 5).

2 “Unknown aetiology” corresponds to the probable cases.

**Figure 7:**
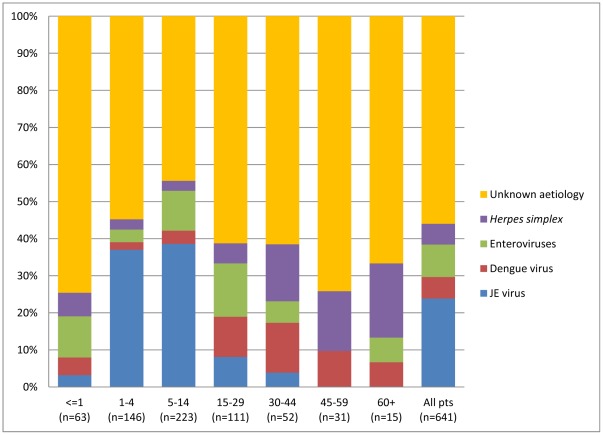
Pathogens of viral encephalitis/meningitis by age group (excluding dual infection cases). Laboratory confirmed aetiology per agegroup (bars) for patients meeting the case definition of viral meningitis/encephalitis (excluding dual infection cases). “Unknown aetiology” corresponds to patients with a diagnosis of probable viral encephalitis/meningitis.

### Aetiology of Bacterial Meningitis

Bacterial pathogens were identified in 198/302 (66%) of adult patients who fulfilled the clinical criteria of BM, by culture or real-time PCR method. *S. suis* serotype 2 caused meningitis in 147/302 (49%) of the adult BM group, which was four times higher than meningitis cases caused by *S. pneumoniae* in this population. *S. suis* was not isolated from paediatric patients. In children, *H. influenzae* type b (39/150, 26%) and *S. pneumoniae* (37/150, 25%) were the main pathogens causing BM (Table 5 and 6). The aetiology of BM varied by age. *H. influenzae* type b was the most common pathogen in children less than 1 year of age, causing 27/66 (41%) of cases. Over 90% (36/39) of *H. influenzae* type b cases were reported in the first five years of life. In adults, *S. suis* serotype 2 caused less than 6/70 (9%) of cases of BM in younger patients (<30 years of age), but was responsible for 141/232 (61%) of BM cases in patients over 30 years old. Twenty five percent of BM cases in children and young adults (56/220) were caused by *S. pneumoniae* but it was identified in less than 10% (16/232) of patients with BM older than 30 years (Figure 6).

### Aetiology of Viral Encephalitis/Meningitis

JE virus was the most common pathogen identified in patients with clinical encephalitis and was responsible for 142/432 (33%) of paediatric VE/M cases. Dengue virus, enteroviruses and *Herpes simplex* virus were the most common identified viral pathogens in adults, each pathogen accounting for 10% of infections (23/209, 20/209 and 22/209, respectively). A viral pathogen was not identified in 56% (359/641) of patients (Table 7). JE virus was responsible for nearly 40% of encephalitis/meningitis cases in the age group 1–14, but was less common in patients younger than 1 year or older than 15 years (Figure 7).

### Tuberculous Meningitis

TBM was responsible for 122/1241 (10%) of cases, including 87/617 (14%) of adult patients and 31/624 (5%) of pediatric patients. Forty nine cases (40%) were confirmed by commercial real-time PCR. Twelve of 122 (10%) patients were classified as probable TBM cases and 61/122 (50%) patients as possible TBM cases using the case definition (see Tables 1, 2 and 3 and 4).

## Discussion

This is the first prospective provincial hospital-based surveillance study on CNS infection in Viet Nam. The participating hospitals function as primary care hospitals for CNS infection patients in their catchment area and therefore the data reflect the burden of disease and associated pathogens within the population. *S. suis* serotype 2 was the predominant microorganism found in adult patients and was four times as common in adult patients as *S. pneumoniae* (49% vs. 12%), the most common cause of BM in Europe and the United States [15], [16]. The results of this study strengthen the results from earlier studies carried out in tertiary referral hospitals in Viet Nam, and in some other intensive pig rearing countries in Asia, in which *S. suis* is the most important pathogen of BM in adult patients [17]–[21]. JE virus, the most common identified cause of paediatric CNS infection, was responsible for 23% (142/624) of paediatric cases. These findings clearly point at the important role of zoonotic infections in Vietnam, given that pigs, the main source of meat in Vietnam, function as the main reservoir for both pathogens.

The culture method was less sensitive than the PCR method in detection of the main pathogens of bacterial meningitis (Table 3, 4, 5 and 6). This may be related to the fact that nearly half of BM patients (236/452) had received antibiotic drugs prior to admission. Hence, the prevalence of other bacterial infections, which were not tested by PCR method, could be possibly underestimated.

We observed seasonality of CNS infections in both adults and children. In the adult population seasonality was driven by *S. suis* serotype 2 infections observed at Hue Central hospital. Hue is located in central Vietnam, where there is a larger fluctuation of air ambient temperature during the year, in comparison with the more stable air temperature of the southern provinces. We have previously shown that ingestion of high risk food items is an important risk factor for *S. suis* infection in Vietnam, in addition to occupational or direct contact due to skin lesions [22]. Bacterial load in contaminated food items is likely to be higher when food is kept at higher ambient temperatures, and this may explain the seasonal pattern observed in Hue. In China and Thailand, more *S. suis* patients are also admitted during the rainy season, between June and September [19], [23]. In the case of JE, epidemiological studies have shown that after the monsoon rains *Culex* mosquitoes, the main vector of JE, breed prolifically with consequent increased disease transmission. In addition, temperature may be an important factor as larval developmental time and extrinsic incubation period of JEV are shorter at higher temperatures [2].

Similar to other studies on CNS infections in Vietnam, the observed ratio between males and females was greater than 1 [3], [24], [25]. In *S. suis* meningitis, male sex is a risk factor related to exposure to pork and pigs, and the ratio of male to female is about 4/1 [22]. For other etiologies we do not have a good explanation for the male dominance.

*H. influenzae* type b (Hib) conjugate vaccine has just been introduced to the Vietnamese National Expanded Program on Immunization (NEPI) since June 2010. Consequently, this pathogen was still the most common cause of BM in children, causing a similar proportion of disease as found in other studies from around the world before the Hib conjugate vaccine era [26]–[30]. The incidence rate of Hib meningitis in Vietnamese children is likely to decrease sharply in the next few years, especially in the age group of children less than 5 years of age. In contrast, conjugate multi-valent *S. pneumoniae* vaccines are not included in the NEPI and JE vaccine is only introduced into NEPI for children 1–5 years of age in the high-risk districts, which are mostly in northern provinces [31].

VE/M offers a considerable diagnostic challenge to physicians. Previous aetiological studies have reported that at least 60% of patients hospitalized with encephalitis had no cause identified despite extensive laboratory testing [25], [32]–[35]. Though our study focused on the common viral pathogens of encephalitis/meningitis in Viet Nam, the proportion of patients with suspected VE/M with no pathogen identified was also approximately 60% of all cases [36]–[38]. JE virus is the most important identified cause of encephalitis in the South-East Asia, being responsible for 24% of VE/M cases with over 90% of cases reported in children aged <15 years in this study. JE rarely occurs in adults who live in endemic regions presumably because of exposure to virus during childhood and subsequent immunity [6]. JE may be more common in the north of Viet Nam than in the central and south of Viet Nam. It caused 217/421 (50%) of acute encephalitis syndrome cases in five northern provinces [31]. Another important arboviral infection in humans, dengue, has become the most important epidemic disease in this country. Neurological manifestations of dengue infection were reported in 0.5–6% of hospitalized dengue hemorrhagic fever patients [37], [39], [40]. In this study, dengue virus, detected by the presence of specific IgM in CSF, was the only pathogen in 39/641 suspected VE/M cases (6.1%), including 25 adults and 14 children. Our result are similar to other studies in the dengue endemic region, where the proportion of dengue virus ranged from 4.2% to 47% of patients with encephalitis [25], [37], [41]–[43]. Dengue should be considered in patients who present with the clinical features of encephalitis, whether or not classical manifestations of dengue are present [37], [44].

In Europe and North America, enteroviruses are among the most common causes of viral meningitis in children [45], [46]. In this study, enteroviruses were the second most common viral pathogen in both adults and children. However, 15/20 adult patients, compared to 6/36 paediatric patients, lived in Thua Thien – Hue province and 14 of these adults, compared to none of the children, were admitted to Hue Central hospital in the period from June to September 2009 (Figure 5). The adult cases observed in our study may therefore be related to an outbreak in Thua Thien – Hue province in the summer of 2009.

Rapid and early diagnosis of CNS infections is fundamental to patient outcome. Physicians face numerous difficulties in establishing a TBM diagnosis, including the often non-specific manifestations, low sensitivity of Ziehl-Neelsen staining and nucleic acid amplification and delays in classical culture methods [47]. To diagnose TBM in this study, we applied the consensus case definition of TBM, which included clinical, CSF, and cerebral imaging information and could be used regardless of patient’s age and HIV status [8]. A commercial real-time PCR test was used to help confirm TBM. Ranking after *S. suis* serotype 2 meningitis and JE, TBM, which was confirmed in 49 (40%) of suspected TBM cases, was the third most common cause of CNS infection in our study. This is the first report of TBM in a context of CNS infection in Vietnamese provincial hospitals. While TBM was the cause of 143/357 (40%) adult suspected CNS infections admitted to HTD [48], the overall TBM proportion and its proportion stratified for adults and children were 10% (122/1241), 15% (91/617) and 5% (31/624), respectively in this study. The proportion of TBM at HTD may be higher because HTD receives patients who fail to respond to treatment with antimicrobial agents for the treatment of BM from provincial hospitals, and these patients are often diagnosed with TBM. In addition, the HIV prevalence may be higher in Ho Chi Minh City and surrounding areas resulting in a higher incidence of TBM. The protective role of BCG vaccination against TBM in children may explain why TBM accounted for a lower proportion of cases in children than in adults [49], [50].

Dual infections in CNS infection, such as among members of herpesviruses or between a member of this group and a bacterium, have previously been reported in both immunocompetent and immunocompromised patients diagnosed using PCR methods and/or intrathecal antibody determinations [51]–[56]. In this study, we found 20 dual infection cases in 1241 suspected CNS infection patients. Most cases (18/20 cases) were infected with an endemic virus (JE virus, dengue virus or enteroviruses) and a bacterium. DENV and JEV are both endemic in south Vietnam and infection is often asymptomatic. Dual infection of JEV and enteroviruses or *H. influenzae* type b and enteroviruses was also reported in other studies in South-East Asia [25], [57]. Our findings emphasize the role of these viruses as a co-infection pathogen in an endemic region and suggest that testing for other pathogens should still be considered when a single pathogen has been identified in the CSF. While the clinical relevance of these co-infections remain unclear, it is tempting to speculate about the interplay between viral and bacterial CNS infections, for example by facilitating entry of one or the other into the CNS compartment [25].

*Cryptococcus neoformans* is one of the most important HIV-related opportunistic infections in South-East Asia, where one third of CNS infection in HIV/AIDS patients are due to this pathogen [58]–[61]. It also causes meningitis in HIV uninfected patients in Viet Nam [62]. Only two cases were reported in our study. This may be explained by the limitation of microbiological culture facilities at the provincial hospitals or by the fact that lumbar punctures were not performed in HIV infected patients at these hospitals. In Viet Nam, attending physicians rarely do lumbar puncture on HIV patients at remote provincial hospitals because of their poor prognosis or relative’s refusal.

CNS infections have a high morbidity and mortality. The case fatality rate (CFR) in this study was 12% in adults and 7% in children, but varied widely according to underlying pathogen, ranging from 5–10% in acute BM and VE/M to 30–40% in TBM. This CFR was likely related to deaths prior to prescription of anti-tuberculous drugs because most TBM patients in Vietnam are admitted first to an infectious diseases unit or a neurology unit in a general hospital before being transferred to a specialist tuberculosis unit or hospital where TBM specific treatment is started. This highlights the critical importance of considering TBM in the differential diagnosis of CNS infection throughout Viet Nam and the need for early diagnosis and treatment.

In conclusion, we identified a cause in 640/1241 (52%) suspected CNS infection cases using molecular diagnostic, serological and culture methods. The three most common pathogens in children were JE virus, *H. influenzae* type b and *S. pneumoniae*. Meanwhile, *S. suis* serotype 2 meningitis, an emerging zoonosis in intensive pig rearing countries, caused one fourth (147/617) of adult suspected CNS infection or a half (147/302) of adult purulent BM in Viet Nam. We also found that case fatality rate of TBM exceeded 30% at the provincial hospitals before TBM diagnosis was established. As patient outcome is directly related to early diagnosis and treatment, physicians should always think of TBM in the context of CNS infection in developing countries.
